# NF-kB and the CLL microenvironment

**DOI:** 10.3389/fonc.2023.1169397

**Published:** 2023-03-30

**Authors:** Alice O’Donnell, Chris Pepper, Simon Mitchell, Andrea Pepper

**Affiliations:** ^1^ Department of Clinical and Experimental Medicine, Brighton and Sussex Medical School, Brighton, United Kingdom; ^2^ Royal Sussex County Hospital, University Hospitals Sussex, Brighton, United Kingdom

**Keywords:** chronic lymphocytic leukemia, NF-kappaB signaling pathway, tumor microenvironment, hematological malignancies, therapeutic targets, CLL

## Abstract

Chronic lymphocytic leukemia (CLL) is the most prevalent type of leukemia in the western world. Despite the positive clinical effects of new targeted therapies, CLL still remains an incurable and refractory disease and resistance to treatments are commonly encountered. The Nuclear Factor-Kappa B (NF-κB) transcription factor has been implicated in the pathology of CLL, with high levels of NF-κB associated with disease progression and drug resistance. This aberrant NF-κB activation can be caused by genetic mutations in the tumor cells and microenvironmental factors, which promote NF-κB signaling. Activation can be induced *via* two distinct pathways, the canonical and non-canonical pathway, which result in tumor cell proliferation, survival and drug resistance. Therefore, understanding how the CLL microenvironment drives NF-κB activation is important for deciphering how CLL cells evade treatment and may aid the development of novel targeting therapeutics. The CLL microenvironment is comprised of various cells, including nurse like cells, mesenchymal stromal cells, follicular dendritic cells and CD4+ T cells. By activating different receptors, including the B cell receptor and CD40, these cells cause overactivity of the canonical and non-canonical NF-κB pathways. Within this review, we will explore the different components of the CLL microenvironment that drive the NF-κB pathway, investigating how this knowledge is being translated in the development of new therapeutics.

## Introduction

Chronic Lymphocytic Leukemia (CLL), is the most common leukemia in the western world. In CLL, monoclonal B-lymphocytes accumulate in the blood, bone marrow and lymph nodes (1). Current treatment strategies vary depending on disease burden, from active monitoring in asymptomatic patients, to targeted therapies in more advanced disease (2). Inhibitors targeting Bruton’s tyrosine kinase (BTK), Phosphoinositide 3-kinases (PI3K) and B cell Lymphoma 2 (BCL2) have revolutionized treatment of CLL, but many patients are refractory or develop resistance, and CLL remains an incurable disease. Approximately 1000 people in the United Kingdom die from CLL each year, so understanding the molecular mechanisms that prevent better treatment of CLL remains an active area of research (3).

One such avenue of research surrounds the role the NF-κB signaling pathway plays in CLL. NF-κB is a transcription regulator, and aberrant activity of its pathways are associated with both inflammatory conditions and malignancies (4–6). Because of this, NF-κB has been identified as a potential therapeutic target for a variety of cancers, including CLL (7). Genetic mutations altering these pathways in CLL are well described (8–10), but over the last decade our understanding of the role of the microenvironment in activating NF-κB has evolved (11). The CLL microenvironment has been implicated in disease progression and chemoresistance; modern therapeutics are often very effective at clearing tumor cells from the peripheral blood but the lymphoid tissues provide a haven for residual disease. Both genetic mutations and the tumor microenvironment are essential in activating NF-κB in CLL, with the latter representing a less well characterized aspect of CLL development that requires further investigation. Herein, we explore the components of the microenvironment which activate NF-κB in CLL, identifying potential therapeutic targets for this prevalent and incurable malignancy.

## The NF-kappaB pathway

The ubiquitous transcription factor NF-κB is actually made up of five different subunits, which act as homodimers or heterodimers to regulate a variety of genes relating to the immune system, inflammation, cell growth and survival (12). The five NF-κB proteins found in human cells are p65 (RelA), RelB, c-Rel, p105/p50, and p100/p52, which work by binding to κB enhancer regions in the genome to control target gene transcription (13). Before activation of the NF-κB pathway, inhibitors called IκBs are coupled with these dimers, rendering them inactive in the cell cytoplasm.

**Figure 1:**
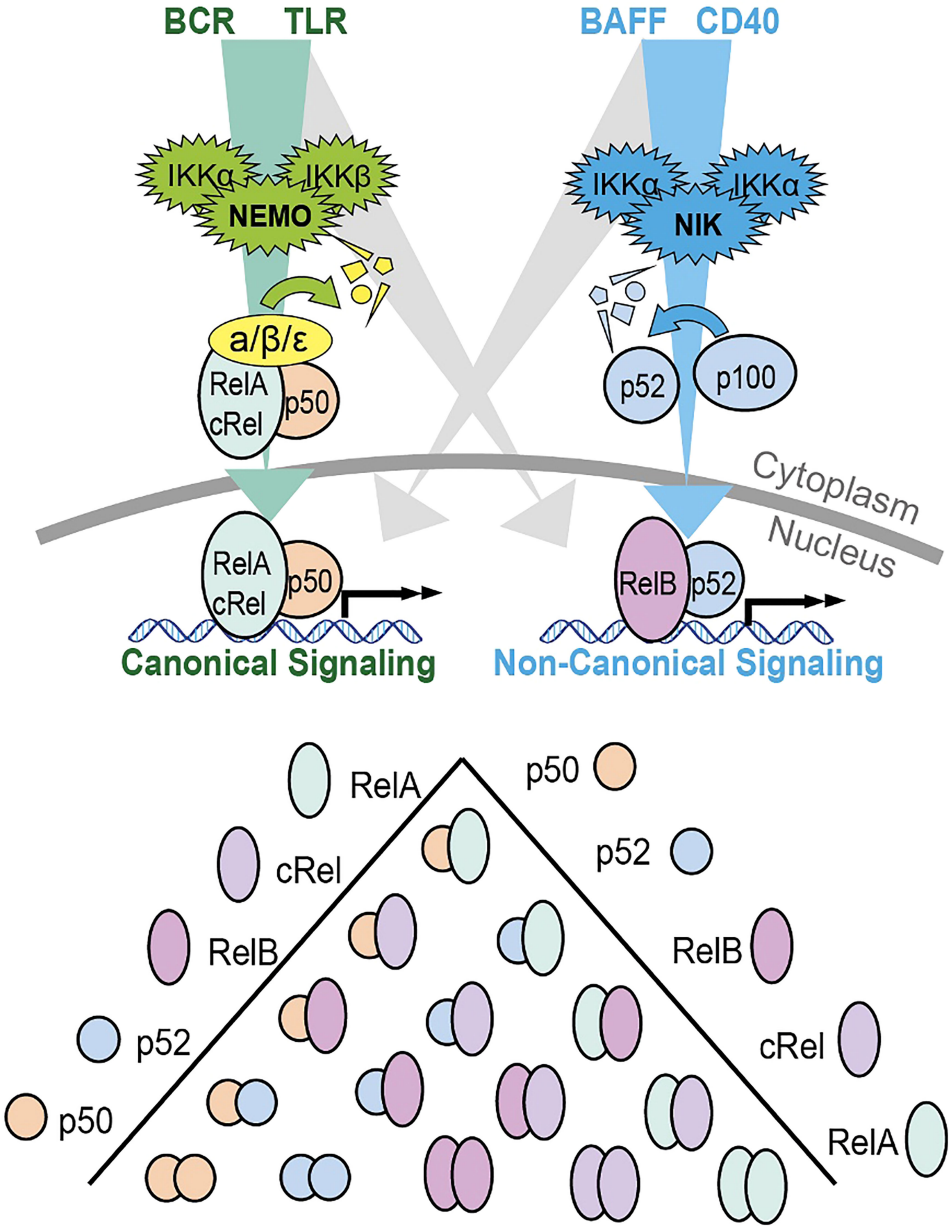
Schematic summary of the canonical and non-canonical NF-κB signaling pathways and the dimers formed by the 5 different subunits. The canonical signaling pathway is shown in green, and primarily induces RelA:p50 and cRel:p50 through the degradation of Inhibitors of NF-κB (IκBs). The non-canonical pathway is indicated in blue and primarily activates RelB:p52 through the processing of p100 into p52. The potential for bi-direction crosstalk between the two pathways is indicated in gray. Both p100 and RelB are target genes on canonical NF-κB signaling creating the potential for crosstalk from canonical to non-canonical signaling. P100 that is not processed into p52 can form an inhibitory complex that inhibits canonical dimers. This p100 can be degraded by NIK creating the potential for crosstalk from non-canonical signaling to canonical dimers. Additional crosstalk can occur due to the competition between p50 and p52 for binding to a limited pool of RelA and RelB.

NF-κB is activated by two pathways: the canonical and non-canonical signaling pathways (**Figure 1**). The canonical pathway is triggered by the binding of ligands to several surface receptors such as the B cell receptor (BCR) and toll-like receptors (TLRs). Such binding activates the IKK trimeric complex, formed of IKKα, IKKβ and IKKγ subunits (14). The IKK complex phosphorylates and degrades IκBα, allowing the translocation of NF-κB subunits, primarily the p50/RelA complex, to the nucleus (15). In contrast, the non-canonical pathway is triggered by activation of different receptors including the B cell activating factor receptor (BAFF-R) and CD40 receptor (16). Ligand induced activation triggers NF-κB inducing kinase (NIK) to phosphorylate the IKKα complex, causing phosphorylation of p100, processing of p100 into p52, and subsequent nuclear translocation of the NF-κB RelB/p52 complex (16). The release and translocation of NF-κB dimers causes transcription of cancer associated target genes such as the anti-apoptotic BCL2 (B cell lymphoma 2), BCL2L1 (B cell lymphoma XL) and MCL1 (Myeloid leukemia cell differentiation protein) and the pro-angiogenic VEGF (vascular endothelial growth factor) gene (17). There is potential for crosstalk between the two NF-κB signaling pathways in B cells (18). Genes that encode non-canonical pathway components p100 and RelB are induced by canonical pathway activity (18), unprocessed p100 can inhibit canonical NF-κB dimers rendering them responsive to non-canonical pathway activity (19), and NF-κB proteins from both pathways compete to form dimers (20). The prevailing direction of crosstalk in non-malignant B cells is non-canonical activity inducing canonical dimers, as canonical pathway activity does not induce RelB:p52 (19, 21). The magnitude and functional significance of this crosstalk in CLL is not known and likely dependent on the microenvironmental context.

Overactivation of the NF-κB pathway has been identified in several conditions, including chronic inflammatory conditions, autoimmune conditions and hematological malignancies (22–24). In CLL, the NF-κB pathway is aberrantly active compared to age-matched healthy B cells, and several recurrent genetic mutations that alter NF-κB activity have been reported (25, 26). Genetic mutations in NOTCH1 are found in approximately 11% of CLL patients (8) and cause increased canonical and non-canonical NF-κB activity (27), which correlate with CLL cell survival and poor response to chemotherapy agents (28–30). Gene expression profiling has shown that NOTCH1 mutated CLL cells have higher expression of genes associated with NF-κB than their wild-type counterparts (31) and these mutations lead to increased NOTCH1 activity and increased nuclear translocation of p65 (RelA). As a result, there is raised expression of NF-κB target genes such as CD49d (32). Xu et al (33) suggested that the crosstalk between NOTCH1 and NF-κB is caused by an increase in intracellular NOTCH1 in mutated CLL cells resulting in amplified nuclear NF-κB DNA binding (34). Other genetic mutations affecting the NF-κB pathway in CLL include: NFKBIE mutations (causing a reduction in the activity of the negative regulator of NF-κB: IκBϵ), BIRC3 mutations (causing an increase in NIK levels) and MYD88 mutations (causing constitutive signaling in the TLR signaling pathway) (35–37). However, genetics alone cannot explain the critical role of NF-κB in the pathology of CLL, so the protective microenvironment that supports CLL cells is an emerging topic of importance (11).

## The CLL microenvironment

**Figure 2:**
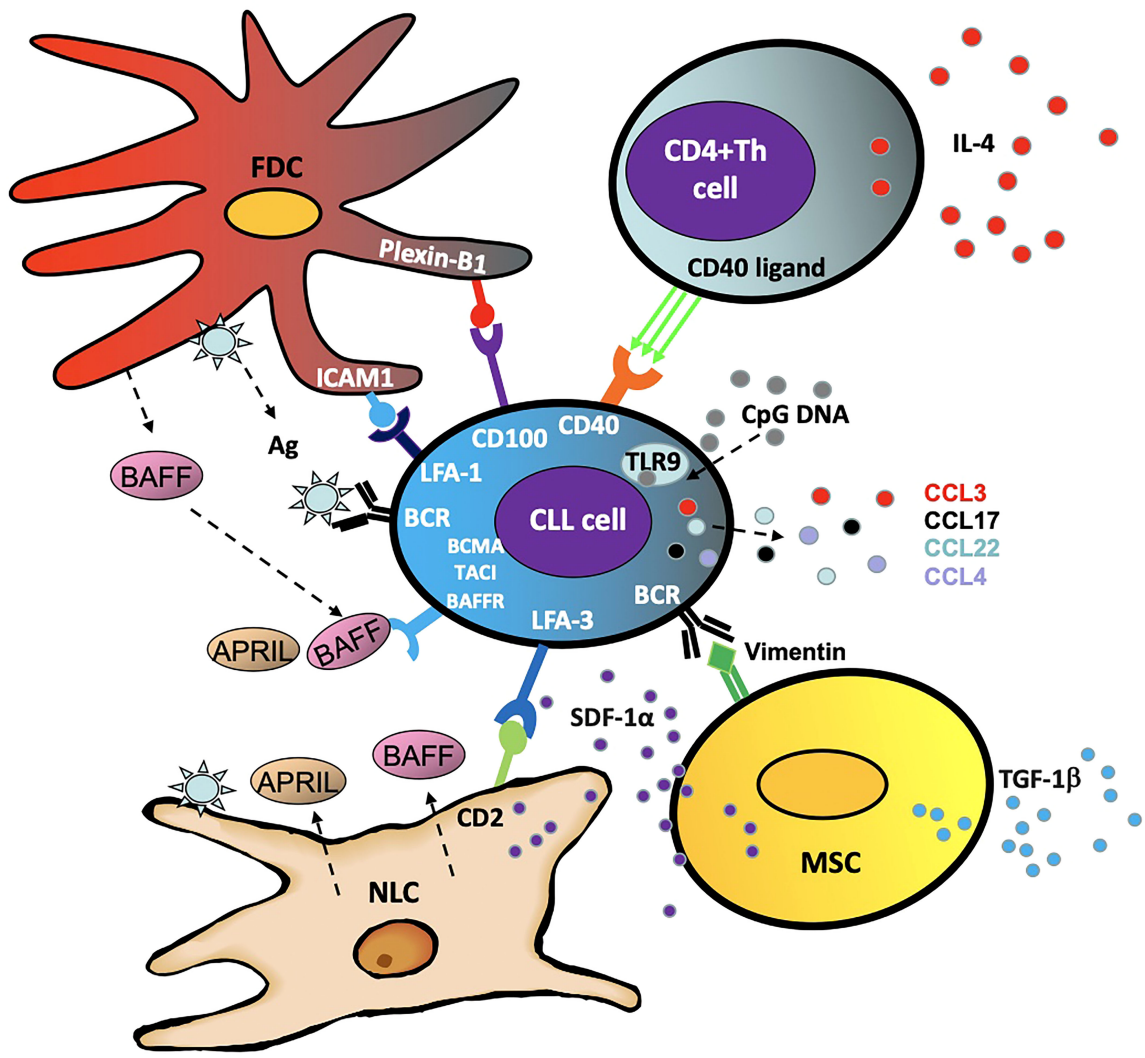
Schematic summary of some of the components and interactions in the CLL microenvironment.

Within the body, CLL cells circulate between the peripheral blood and lymphoid organs where they receive survival and proliferation signals. Within the lymph node, they are also more resistant to therapeutic destruction and depend largely on surrounding stromal and T cells, which comprise the CLL ‘microenvironment’ (**Figure 2**) (38). Interactions with this microenvironment promotes cell division and tumor cell survival (39). Furthermore, activated CLL cells produce chemo-attractants such as CCL3, CCL4, CCL17 and CCL22 in order to draw in these supportive cells and facilitate crosstalk between the CLL cell and its microenvironment (40). The following are some of the cells that interact with and support CLL cells by activating the NF-κB pathway:

### Nurse like cells

Nurse-like cells (NLC) represent a population of leukemia-associated macrophages expressing CD14, CD45, HLA-DR, CD33, and CD68, which are induced by CLL cells through nicotinamide phosphoribosyl transferase (NAMPT) and high-mobility group protein B1 (HMGB1) signaling (41–43). In addition to their ability to establish firm contact and support CLL survival through stromal-derived factor 1-α (SDF-1α) and lymphocyte function-associated antigen 3 (LFA-3), these cells cause overactivation of both the canonical and non-canonical NF-κB pathway *via* several mechanisms (43). Primarily these include the release of B cell activation factor (BAFF), a proliferation-inducing ligand (APRIL) and B cell receptor (BCR) signaling *via* antigen dependent and independent mechanisms (44).

### Mesenchymal stromal cells

The multipotent mesenchymal stromal cells (MSC) form part of the CLL protective microenvironment (45) and are characterized by CD73, CD90 and CD105 expression (46). Part of MSC's ability to encourage CLL cell survival is independent of the NF-κB pathway, with transforming growth factor β1 (TGFβ1) and stromal cell derived factor 1 (SDF1) driving CLL cell survival (45). However, by activating the BCR through ligands such as calreticulin and vimentin, the MSCs cause aberrant canonical NF-κB activity in the CLL cell, promoting survival and proliferation (47).

### Follicular dendritic cells

Located in the secondary lymphoid organs and bone marrow in CLL, follicular dendritic cells (FDCs) also provide support for the CLL cells (48, 49). Expressing high levels of complement receptors CR1 and CR2, these cells crosstalk with CLL cells *via* NF-κB independent interactions such as plexin-B1/CD100 and ICAM1/LFA-1 (intracellular adhesion molecule 1/lymphocyte function-associated antigen 1) (50, 51). Crucially, in a similar fashion to the MSCs, FDCs trigger activation of NF-κB through the BCR and BAFF in order to nurture and support the CLL cells (52, 53).

### CD4+ T cells

CD4+ helper T cells are a key component of the adaptive immune system which contribute to the CLL microenvironment (54). The presence of CD4+ T cells is essential for CLL development, as exemplified by the inability of CLL cells to proliferate in mice lacking CD4+ T cells (55). CD4+ T cells increase STAT6 driven BCR signaling through release of IL4 (56) and in normal B cells, this has been shown to be *via* activation of the non-canonical NF-κB pathway (57). CD4+ T cell activation of the non-canonical NF-κB pathway in CLL is also through CD40L activation of CD40 (58, 59) and, as a result, CD40L and IL4 are important components of laboratory-based co-culture systems which aim to mimic the tumor microenvironment (40, 60, 61).

## Microenvironmental activation of the NF-κB pathway

The above components of the microenvironment act on the CLL cell through several receptors, posing exciting potential therapeutic targets for this disease. Below are the key receptors involved in activating the NF-κB pathway in this malignancy:

### B cell receptor (BCR) signaling

The BCR is a transmembrane receptor located on the surface of B cells, and is comprised of the co-receptors CD79A and CD79B, along with the membrane bound immunoglobulin molecule (sIgM) (62). After ligand binding to the BCR, the Src family kinases Lyn and spleen tyrosine kinase (SYK) phosphorylate the cytoplasmic segment of the CD79A and CD79B heterodimers. As a result, several adaptor proteins and kinases are phosphorylated and activated, including BTK, PI3Kδ and phospholipase Cγ2 PLCγ2 (63). Activation of BTK induces the canonical NF-κB pathway, with downstream degradation of IκBs leading to NF-κB translocation and target gene transcription (63, 64).

BCR signaling is increased in CLL cells compared to normal B cells, with CLL cells also expressing higher levels of LYN, SYK and BTK than non-malignant B cells (65). Gene expression profiling has shown that BCR signaling, canonical NF-κB activation and proliferation are all up-regulated in lymph node resident CLL cells compared to those in the peripheral blood (58). Autonomous antigen-independent signaling is one way in which the BCR is activated in CLL, involving binding of an internal epitope to the heavy-chain complementarity-determining region (HCDR3) of the BCR (66). In addition to autonomous BCR signaling, external activation of the BCR through the CLL microenvironment has been suggested, with stroma cell expression of antigens such as vimentin and calreticulin which activate the BCR (47). In addition to stimulating the BCR directly, cytokines within the microenvironment, such as IL4 and IL6, have been found to upregulate sIgM in CLL *in vitro* (67). NF-κB activation as a result of microenvironmental activation of BCR signaling enables the proliferation and survival of the CLL cells.

The importance of the BCR in the CLL microenvironment is shown by the revolutionary effects of phosphatidylinositol 3 kinase (PI3K) δ inhibitors such as idelalisib and duvelisib and BTK inhibitors, such as ibrutinib, acalabrutinib and zanubrutinib (68). Crucially, both PI3Kδ and BTK inhibitors exhibit their clinical effects by causing redistribution of CLL cells out of the lymph nodes into the peripheral blood, demonstrating the importance of both in CLL motility and retention (69, 70). In addition to preventing canonical NF-κB activation through targeting the BCR *via* BTK, ibrutinib treatment also induces loss of NLC mediated pro-survival signaling in the microenvironment (71). Moreover, inhibition of canonical NF-κB activation through targeting BTK has been found to reduce CD4+ and CD8+ T cells within the microenvironment and dampen expression of chemo-attractants produced by tumor associated macrophages (71–73). Furthermore, BTK inhibition reduces CXCR4-mediated signaling and adhesion, releasing CLL cells into the circulation and preventing them from re-entering the CLL protective niche *in vivo* (74).

### Toll-like receptor (TLR) signaling

The TLRs are transmembrane glycoproteins, composed of a C-terminal domain, a transmembrane domain and an N-terminal domain, which are expressed in several cells including B cells (75). There are 10 functional TLRs in humans, which can either be expressed on the cell surface or within the endosomes, and they trigger the NF-κB through two routes. On activation, the TLR recruits the TIR domain adaptor protein MyD88 (76). MyD88 subsequently interacts with members of the IL1 receptor-associated kinase (IRAK) family, with activation and phosphorylation of IRAK1 and IRAK4 causing downstream recruitment of transforming growth factor-β-activated kinase-1 (TAK1) (77). TAK1 is also activated when TLR activation leads to recruitment of TRIF, activating the IKK complex and thus, the NF-κB pathway (78).

To date, there is very little *in-vivo* data to support a role for TLR in the pathology of CLL. Martines et al. (79) reported that CLL proliferation is dependent on BCR and macrophage derived signals as opposed to TLR in the murine Eμ-TCL1 model of CLL (79). However, crosstalk between the TLR and BCR pathways (80) and formation of a supercomplex formed of MyD88, TLR9, and the BCR (My-T-BCR) (81) has been documented in aggressive lymphoma and indicate that these pathways cannot be considered in isolation. Furthermore, neutrophil extracellular traps have been shown to directly upregulate TLR9 signaling in DLBCL and subsequently activated NF-κB, STAT3, and p38 pathways to promote tumor progression (82). Activation of TLR signaling has been reported in lymph node resident CLL cells (83) and *in-vitro* models have suggested that TLR signaling and subsequent NF-κB activation is associated with an increase in CLL proliferation and survival, and with resistance to chemotherapy (84, 85).

In CLL patients, TLR1, 2, 6 and 10 are found on the cell surface, with TLR7, 8 and 9 expressed on intracellular endosomes (84). In particular, TLR9 has been highlighted as an important receptor in the CLL microenvironment, with CLL cells demonstrating increased levels of TLR9 compared to normal B cells (86). TLR9 expression *in vitro* has been linked with increased levels of CLL cell migration, while CLL patients have been found to have significantly higher plasma levels of the TLR9 ligand - unmethylated cytosine guanine dinucleotide (CpG)-DNA, compared to healthy controls (87). TLR9 signaling has also been implicated as a potential resistance mechanism to ibrutinib and venetoclax through NF-κB driven upregulation of MCL-1 and BCL-XL (87, 88). Furthermore, IRAK4 and IRAK1, downstream components of the TLR signaling pathway, have been suggested as potential therapeutic targets for CLL (89, 90). These *in-vitro* findings implicate TLR9 as a potential driver of NF-κB in the CLL microenvironment with the potential to increase CLL cell activation and migration, even in the presence of BTK and BCL2 inhibition. However, the clear discrepancy between *in-vivo* and *in-vitro* data supports the need for more studies in this area.

### B cell activating factor (BAFF)

As members of the tumor necrosis factor (TNF) group, BAFF and APRIL regulate the function of the B cell through the activation of the following receptors: B cell maturation antigen (BCMA); Transmembrane activator or the calcium modulator and cyclophilin ligand-interactor (TACI) and the B cell activating factor receptor (BAFFR) (91). The latter is key in activating the pathway, with BAFF/BAFFR ligation leading to the recruitment and degradation of TRAF3 and subsequently the TRAF/cIAP complex, releasing NIK and activating the non-canonical NF-κB pathway (92). BAFF and APRIL also support CLL survival through the canonical pathway, with receptor ligation leading to downstream degradation of IκBα (52). *In vitro* studies have demonstrated that malignant B cells in CLL express BAFF and APRIL receptors, and when stimulated these receptors enhance CLL cell survival (93).

Within the CLL microenvironment, NLCs and FDCs have been found to express increased levels of BAFF and APRIL, with these ligands inducing NF-κB pathway activation (52, 94). *In vivo*, the Eμ-TCL1 CLL mouse model crossed with stromal cell-expressing BAFF transgenic mice showed early progression and decreased survival (95). Indeed, BAFF has been identified as a component of the microenvironment that is able to protect CLL cells from NF-κB inhibition-induced apoptosis (96). The importance of BAFF in the context of CLL cell survival is also evident in the clinical context, with patients expressing low serum levels of BAFF showing better overall survival than those with high serum BAFF levels (97).

Given its role in CLL cell survival, BAFF is a potentially attractive therapeutic target. Antibodies targeting the BAFFR lead to increased levels of BAFF-mediated apoptosis, and also improve the efficacy of ibrutinib *in vivo* (98). Another therapeutic which primarily targets PI3K and histone deacetylase (HDAC) has been shown to work in part by reducing BAFF and APRIL-mediated NF-κB signaling, with the authors highlighting the importance of targeting these cytokines as crucial microenvironmental factors (99). Intriguingly, a phase 2 clinical trial using Belimumab, an anti-BAFF monoclonal antibody, in combination with Rituximab/Venetoclax is underway, with preclinical findings showing promising results (100, 101).

### CD40 signaling

A membrane receptor which is present on various hematopoietic and stromal cells, CD40 binds to its ligand CD40L (upregulated on activated T cells) causing various effects, including germinal center formation, cell survival and cytokine production (102). Through the action of TRAFs, CD40/CD40L binding activates the canonical and non-canonical pathway in B cells, and in CLL has induced NF-κB mediated survival (103, 104). In one arm, CD40/40L binding leads to the activation of TRAF2 and TRAF6, causing downstream activation of the canonical NF-κB pathway (104). In contrast, CD40/CD40L recruits TRAF2 and TRAF3, causing activation of the non-canonical NF-κB pathway through downstream activation of NIK (105, 106).

Within the CLL microenvironment, CD40 stimulation by CD40L-expressing CD4+ T cells activates CLL B cells and contributes to cell proliferation and protection from apoptosis *via* the NF-κB pathway (107, 108). These CD4+ T cells are recruited into the lymphoid niche by chemokines secreted by CLL cells, and the interplay between malignant B cells and CD4+ T cells leads to disease progression (109). In addition, stimulating the CLL BCR causes an upregulation of CD40, while CD40 stimulation has been shown to activate the BCR signaling pathway, suggesting important crosstalk between the BCR and CD40 in CLL cell survival (110, 111).

In addition to contributing to CLL cell survival, CD40L has been implicated in drug resistance in CLL, with microenvironmental agonists including CD40L inducing NF-κB mediated resistance to Venetoclax and ibrutinib (88, 112, 113). Interestingly, direct inhibition of NIK *in vitro* overcomes the protection offered by the CD40/CD40L interaction and induces CLL cell apoptosis in previously resistant cells (59). Targeting CD40/CD40L signaling has also been investigated in the context of CLL, with monoclonal antibody to CD40 Selicrelumab showing promising results through the sensitization of CD20 monoclonal antibodies such as Rituximab and Obinutuzumab (114, 115). Indeed, monoclonal antibodies targeting CD40 such as Dacetuzumab have been investigated in phase 1 and 2 clinical trials for conditions such as multiple myeloma and diffuse large B cell lymphoma (DLBCL), perhaps paving the way for a new therapeutic for other hematological malignancies such as CLL (116).

## Conclusion

In conclusion, there are multiple avenues in which the CLL microenvironment protects the malignant B cells and encourages proliferation *via* the NF-κB pathway. In contrast to a one-way model in which the protective niche supports proliferating cells in isolation, a bi-directional model exists in which CLL cells also recruit and support cells of the microenvironment in a harmonious fashion. A variety of stromal and hematopoietic cells contribute to the microenvironment, and through a variety of receptors including the BCR, BAFF, TLR and CD40, the NF-κB pathway is overactivated. Given its importance in CLL, these components are exciting therapeutic targets and preclinical data suggests an emerging role for these novel therapeutic approaches.

## Author contributions

AO: project administration, study design, literature search, data synthesis, literature appraisal, writing of initial draft. CP: data interpretation and analysis, critical revision and editing of draft. SM: data interpretation and analysis, critical revision and editing of draft, preparation of figures. AP: conception of study, critical review and editing of draft, funding acquisition, supervision of project. All authors contributed to the article and approved the submitted version.
